# Macrophage Phenotype Is Associated with Disease Severity in Preterm Infants with Chronic Lung Disease

**DOI:** 10.1371/journal.pone.0103059

**Published:** 2014-08-12

**Authors:** Lynne R. Prince, Nicola C. Maxwell, Sharonjit K. Gill, David H. Dockrell, Ian Sabroe, Eamon P. McGreal, Sailesh Kotecha, Moira K. Whyte

**Affiliations:** 1 Academic Unit of Respiratory Medicine, Department of Infection and Immunity, University of Sheffield, Sheffield Teaching Hospitals NHS Trust, Sheffield, United Kingdom; 2 Academic Unit of Immunology and Infectious Disease, Department of Infection and Immunity, University of Sheffield, Sheffield Teaching Hospitals NHS Trust, Sheffield, United Kingdom; 3 Department of Child Health, Cardiff University, Cardiff, United Kingdom; Helmholtz Zentrum München/Ludwig-Maximilians-University Munich, Germany

## Abstract

**Background:**

The etiology of persistent lung inflammation in preterm infants with chronic lung disease of prematurity (CLD) is poorly characterized, hampering efforts to stratify prognosis and treatment. Airway macrophages are important innate immune cells with roles in both the induction and resolution of tissue inflammation.

**Objectives:**

To investigate airway innate immune cellular phenotypes in preterm infants with respiratory distress syndrome (RDS) or CLD.

**Methods:**

Bronchoalveolar lavage (BAL) fluid was obtained from term and preterm infants requiring mechanical ventilation. BAL cells were phenotyped by flow cytometry.

**Results:**

Preterm birth was associated with an increase in the proportion of non-classical CD14+/CD16+ monocytes on the day of delivery (58.9±5.8% of total mononuclear cells in preterm vs 33.0±6.1% in term infants, p = 0.02). Infants with RDS were born with significantly more CD36^+^ macrophages compared with the CLD group (70.3±5.3% in RDS vs 37.6±8.9% in control, p = 0.02). At day 3, infants born at a low gestational age are more likely to have greater numbers of CD14^+^ mononuclear phagocytes in the airway (p = 0.03), but fewer of these cells are functionally polarized as assessed by HLA-DR (p = 0.05) or CD36 (p = 0.05) positivity, suggesting increased recruitment of monocytes or a failure to mature these cells in the lung.

**Conclusions:**

These findings suggest that macrophage polarization may be affected by gestational maturity, that more immature macrophage phenotypes may be associated with the progression of RDS to CLD and that phenotyping mononuclear cells in BAL could predict disease outcome.

## Introduction

Chronic lung disease (CLD) of prematurity, often also called bronchopulmonary dysplasia (BPD), is a significant complication of preterm birth, leading to increased respiratory symptoms, repeated hospital admissions and abnormal long term lung physiology resulting in great economic cost and markedly increased parental burden. The pathogenesis of CLD is linked to a number of clinical factors, including prematurity, mechanical ventilation, oxygen therapy and post- and ante-natal infection all of which help to initiate or sustain an inflammatory process in the preterm lung. Persistent airway neutrophilia and elevated levels of neutrophil chemoattractants, including CXCL8, in broncho-alveolar lavage (BAL) fluid, are associated with the development of CLD in preterm infants [1]. Inappropriate suppression of neutrophil apoptosis is also associated with progression to CLD in preterm infants [2]. Macrophages play important roles in inducing and resolving neutrophilic inflammation but their role in CLD is not well defined, particularly in view of the difficulty in acquiring samples from significantly preterm infants. Unresolved questions are how the preterm infant lung orchestrates this inflammatory response and the role of macrophage populations in this process. The limitations in our understanding of lung injury pathogenesis in preterm infants limits our ability to predict which infants may go onto to experience dysregulated inflammation and develop CLD, and also our ability to develop targeted interventions to improve outcome.

Our previous results have demonstrated increased numbers of macrophages in infants with respiratory distress syndrome (RDS), a condition associated with more effective resolution of inflammation and better clinical outcome than CLD, which is characterized by chronic distal airway inflammation and poor lung function. This observation, together with the known roles of macrophages, suggests macrophages may regulate inflammatory responses in the preterm lung [2]. This could derive from a number of the known roles of differentiated macrophages, alone or in combination, including the surface expression of death receptors ligands that may initiate apoptosis in vivo, the production of anti-inflammatory cytokines such as IL-10, or via efferocytosis and cell clearance [3], [4], [5], [6], [7].

These data led us to hypothesise that the relative abundance of macrophages in the preterm lung, and their differentiation status and activation phenotypes, may be associated with either the resolution of RDS or the progression to CLD. In this study, macrophages in BAL fluid samples from preterm infants retrospectively diagnosed with RDS or CLD, and from infants born at term, were phenotyped by flow cytometry, and the relationships between macrophage phenotype, disease severity and gestational age were examined.

## Materials and Methods

Patients were recruited in the regional neonatal intensive care unit at the University Hospital of Wales, Cardiff. The study was approved by the Cardiff and Vale NHS Trust Research and Development Committee and the South East Wales Research and Ethics Committee (05/WSE04/85). Written informed consent was obtained from the parents.

Two groups of infants were recruited: 1) Preterm infants (≤32 weeks gestation) who required mechanical ventilation for RDS defined by clinical and radiological features, were recruited within the first 12 hours of life (n = 18). This group was later subdivided into those infants who went on to develop CLD (oxygen dependence at 36 corrected gestation, n = 11) and those infants where RDS resolved (RDS group, n = 7).

2) Term control infants (>37 weeks gestation, n = 4) who required mechanical ventilation for non-respiratory reasons (for example infants ventilated peri-operatively for gastroschisis).

Bronchoalveolar lavage (BAL) was performed within 24 hours of birth on ventilated infants as previously described [8], [9]. Briefly, with the infant lying supine and the head turned to the left, a size 6 French Gauge (FG) catheter was introduced down the endotracheal tube until resistance was felt, then 1 ml/kg of 0.9% saline (maximum of 2 ml) was instilled via the catheter. The catheter was connected to 8–12 kPa of suction pressure and the lavage fluid collected in a suction trap as the catheter was withdrawn. The procedure was repeated and the lavages pooled for immediate analysis.

Cellular pellets were separated by centrifugation and mucus disrupted by treatment with dithiothreitol (DTT). DTT had no effect on antigen detection by any antibody used (data not shown). A cell count was performed and cytospins prepared (Shandon, Thermo Fisher, Loughborough, UK) for morphological analysis. Slides were air dried, fixed in methanol and stained with Giemsa based stains (Diff-Quik, Reagena, Toivala, Finland).

### Flow cytometry

Cells were washed to remove DTT and stained with the indicated antibodies: HLA-DR-APC (BD Bioscience, Oxford, UK), CD16-PE, CD36-PE, CD15-APC (Biolegend, San Diego, CA) and CD14-biotin (Southern Biotech, Birmingham, AL). Staining combinations were as follows: CD15, CD14/CD16, CD14/HLA-DR/CD36. Streptavidin-PE-Cy5.5 tandem conjugate (eBioscience) was used as a secondary reagent to detect biotinylated primary antibodies. Following staining, samples were immediately analysed by flow cytometry (FACScalibur, Becton Dickinson, Oxford, UK).

Neutrophils were identified as CD15^+^ events. Cells of the monocyte/macrophage lineage were identified as CD14^+^. CD15^+^ neutrophils were CD14 negative, allowing distinction of these two cell types [10]. CD14^+^/16^++^ non-classical monocytes were identified as highly CD16 positive CD14^+^ cells. Forward scatter (FSC) and side scatter (SSC) profiles were also examined. Flow cytometry data were analysed using FlowJo (Tree Star Inc, Ashland, OR).

### Cytokine bead array (CBA) analysis

Cytokines were measured in cell-free BAL using a CBA custom flex set multiplexed assay (BD Biosciences). Antibodies to IL-1β, CXCL8, CCL3, CCL4 (pro-inflammatory indicators) and IL-10 (anti-inflammatory indicator) were used and samples analyzed by FACS array (BD Biosciences).

### Statistical analysis

Data were analysed by Mann Whitney test and regression analysis by linear regression (GraphPad Prism, La Jolla, CA) as defined in the figure legend.

## Results

**Figure 1:**
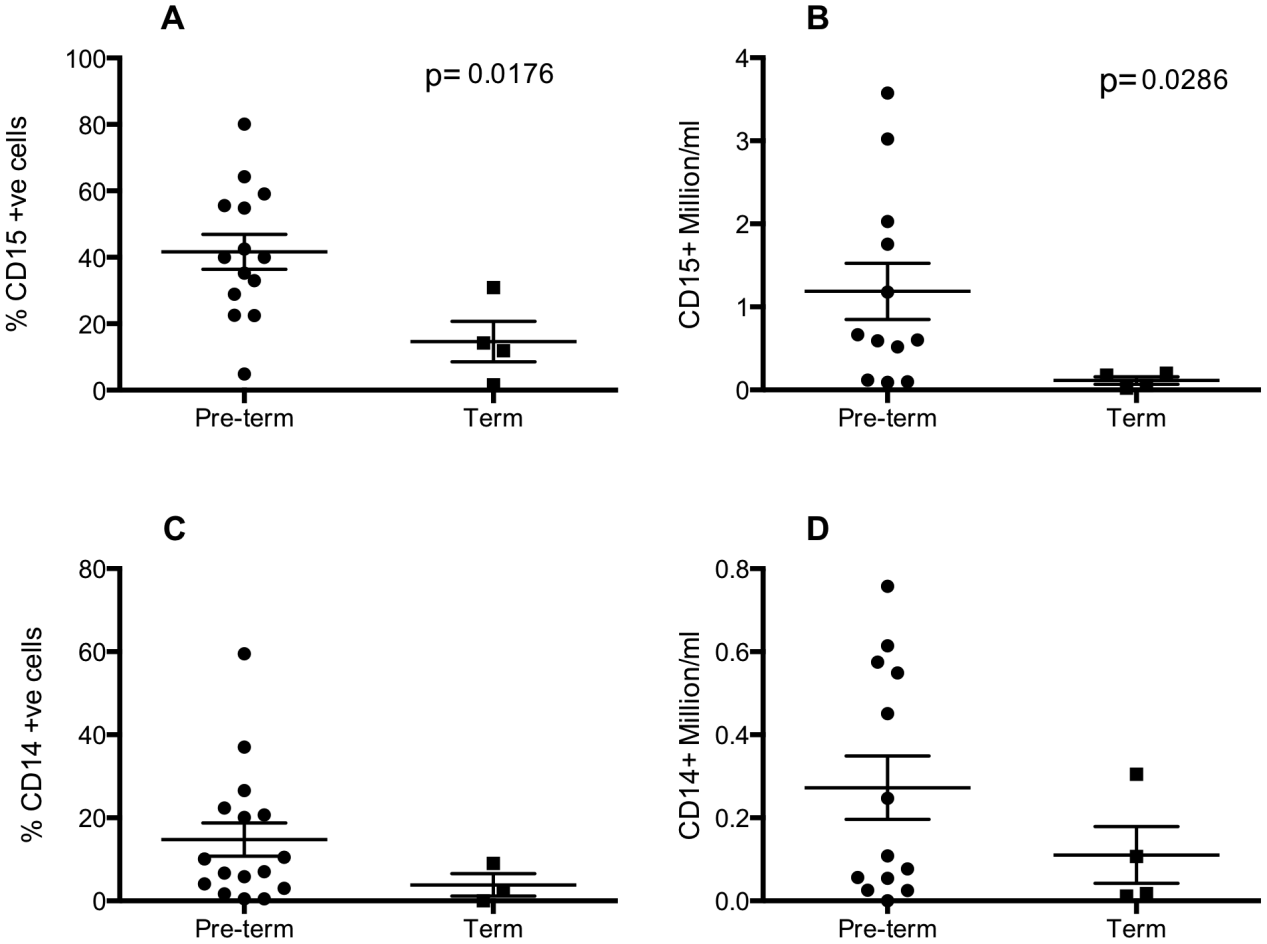
Preterm delivery is associated with increased neutrophils and monocyte–macrophages in the airway. Infants were ventilated and lavaged within 24-CD14, anti-CD15 or matched isotype antibodies and analysed by flow cytometry. Neutrophils (A, B) are defined as CD15+ events and macrophages (C, D) are defined as CD14+ve events and expressed as percent of all cells (A, C) or as absolute numbers/ml lavage fluid (B, D). Statistical analyses were carried out by Mann Whitney test and compared samples collected from pre-term (n = 12–16 of which RDS∶CLD 6–8∶8) to term (n = 4) infants.

**Figure 2:**
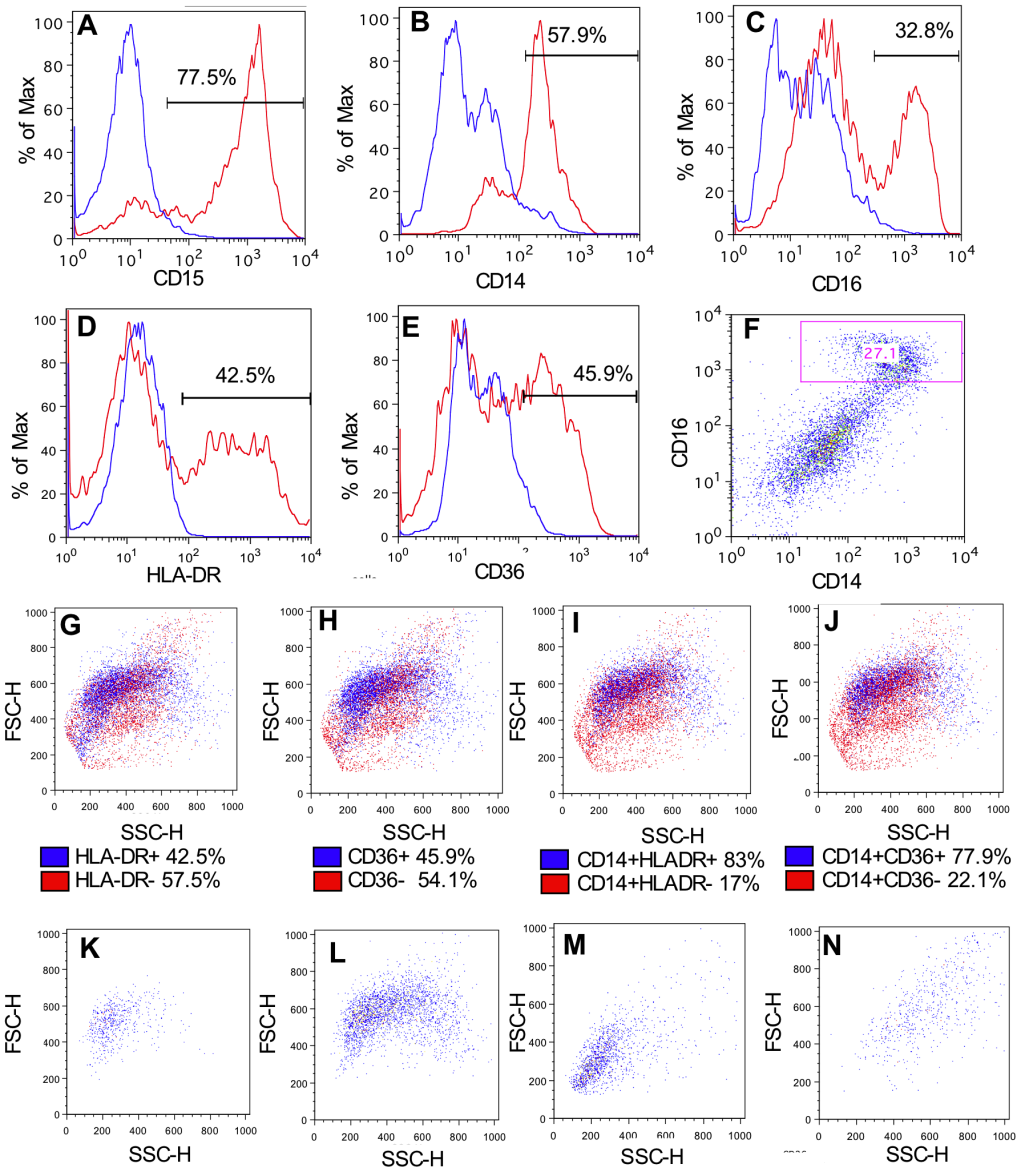
Flow cytometry strategy for identifying functionally polarised macrophage populations in BALF. Infants were ventilated and lavaged within 24-CD15 (A), anti-CD14 (B), anti-CD16 (C), anti-HLADR (D), anti-CD36 (E) or matched isotype antibodies and analysed by flow cytometry. Staining profiles showing isotype controls in blue and target antibody in red. CD14/CD16 staining profile shown in F. Positively stained events are higlighted in FSC and SSC plots in blue for HLADR (G), CD36 (H), CD14/HLADR dual stained (I) and CD14/CD36 dual stained (J). FSC/SSC profiles of CD14+ and: HLADR− (K), HLADR+ (L), CD36− (M) and CD36+ (N) events. Plots illustrate representative data from 3 individual subjects.

Characteristics of the 22 infants included in this study are shown in Table 1. The cellularity of BAL was studied by flow cytometry using antibodies to CD15, CD14, CD16, CD36 and HLA-DR. Mean±SEM numbers of cells (×10^6^) recovered/ml were 1.02±0.29 (term), 4.17±1.78 (RDS) and 1.42±0.2.86 (CLD). The numbers of neutrophils (CD15^+^) recovered were significantly higher in preterm infants compared with term infants within 24 hours of birth, both as a percentage of all cells and as absolute numbers (figure 1A, B). CD14, a co-receptor for TLR signaling, is expressed by both airway macrophages and monocytes [11]. CD14^+^ cells were greater in both percentage and absolute number (figure 1C, D) in some preterm compared with term infants, although differences were not statistically significant. Figure 2 shows flow cytometry plots of samples stained with antibodies to CD15, CD14, CD16, CD36, HLA-DR and respective isotype controls (panels A–E). A box is drawn around CD14^+^/CD16^++^ cells to illustrate gating strategy used (figure 2F). Since CD14 is downregulated as macrophages mature [3], macrophage numbers were assessed both by CD14 and HLA-DR or CD36 co-expression and as HLA-DR/CD36 single stained events, the latter to account for CD14 low macrophages (figure 2D–E & G–H). Cells single stained for HLA-DR or CD36 (figure 2G, H) appear in the same region as cells dual positive for CD14^+^ and either HLA-DR (figure 2I) or CD36 (figure 2J). Since both gating strategies identified cells in the same FSC/SSC region and numbers of cells were comparable for both approaches, a single approach of CD14/CD36 or CD14/HLA-DR positivity was followed for the remaining analysis. HLA-DR^−^/CD36^−^ events were identified in a SSC/FSC low region (indicating cells are smaller and less granular) while HLA-DR+/CD36^+^ events were characterized as mid FSC and mid-high SSC (indicating cells are larger and more granular, figure 2K–N). HLA-DR^+^/CD14^−^ events were gated as dendritic cells, although very low numbers were identified in all samples (data not shown).

**Table 1:** Patient information.

	Preterm group	Term controls
**Number of Patients**	18	4
**Number of Samples**	30	6
**Median Gestational Age (range)**	26^+5^ (23^+4^–31^+2^ wks)	38^+1^ (37^+2^–38^+3^ wks)
**Median Birth Weight (range)**	960 g (550–1920 g)	2730 g (2570–3000 g)
**Male∶Female**	11∶7	4∶0
**Vaginal∶Caesarean delivery**	10∶8	1∶3
**RDS∶CLD**	7∶11	-
**At least 1 dose antenatal corticosteroids**	14/18 (78%)	1/4 (25%)
**Patent Ductus Arteriosus**	14/18 (78%)	0/4 (0)%
**Surfactant therapy (%)**	18/18 (100%)	0/4 (0%)
**Rupture of membranes >24 hours**	4/18 (22%)	0/4 (0%)
**Median days of ventilation (range)**	18 (1–92 d)	3.5 (2–21 d)

**Figure 3:**
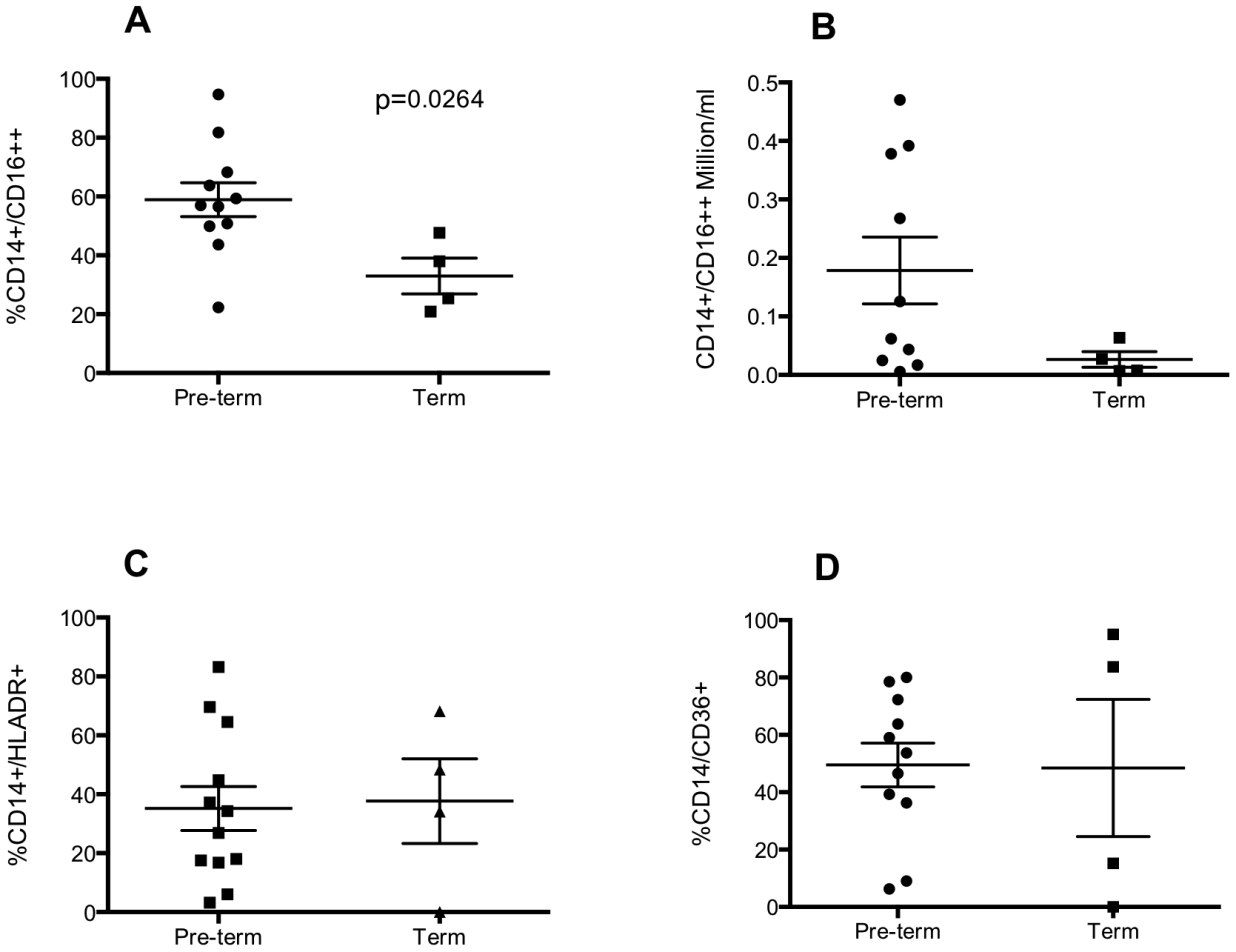
Preterm delivery is associated with increased non-classical macrophages in the airway. Infants were ventilated and lavaged within 24-CD14, anti-CD16, anti-CD36, anti-HLADR or matched isotype antibodies and analysed by flow cytometry. Non-classical macrophages are defined as CD14+/CD16++ and expressed as percent of all macrophages (A) or absolute numbers of CD14+/CD16++ cells/ml lavage (B). CD14+/HLA-DR+ and CD14+/CD36+ cells are expressed as percent of all macrophages (C, D). Statistical analyses were carried out by Mann Whitney test and compared samples collected from pre-term (n = 10–12 of which CLD∶RDS; 7–8∶3–4) to term (n = 4) infants.

Elevated numbers of non-classical monocytes (CD14^+^/16^++^) are associated with inflammation and injury [12] and are also a source of alveolar macrophages [13]. As a proportion of CD14^+^ cells, the proportion of CD14^+^/16^++^ cells was significantly greater in preterm infants compared with term infants at on the within 24 hours of birth (figure 3A). Although a trend for increased absolute number of CD14^+^/16^++^ in preterms is seen, these data do not reach statistical significance (figure 3B). As a proportion of all CD14^+^ cells, there is no significant difference between either HLA-DR^+^ or CD36^+^ macrophages in preterm or term samples (figure 3C, D).

**Figure 4:**
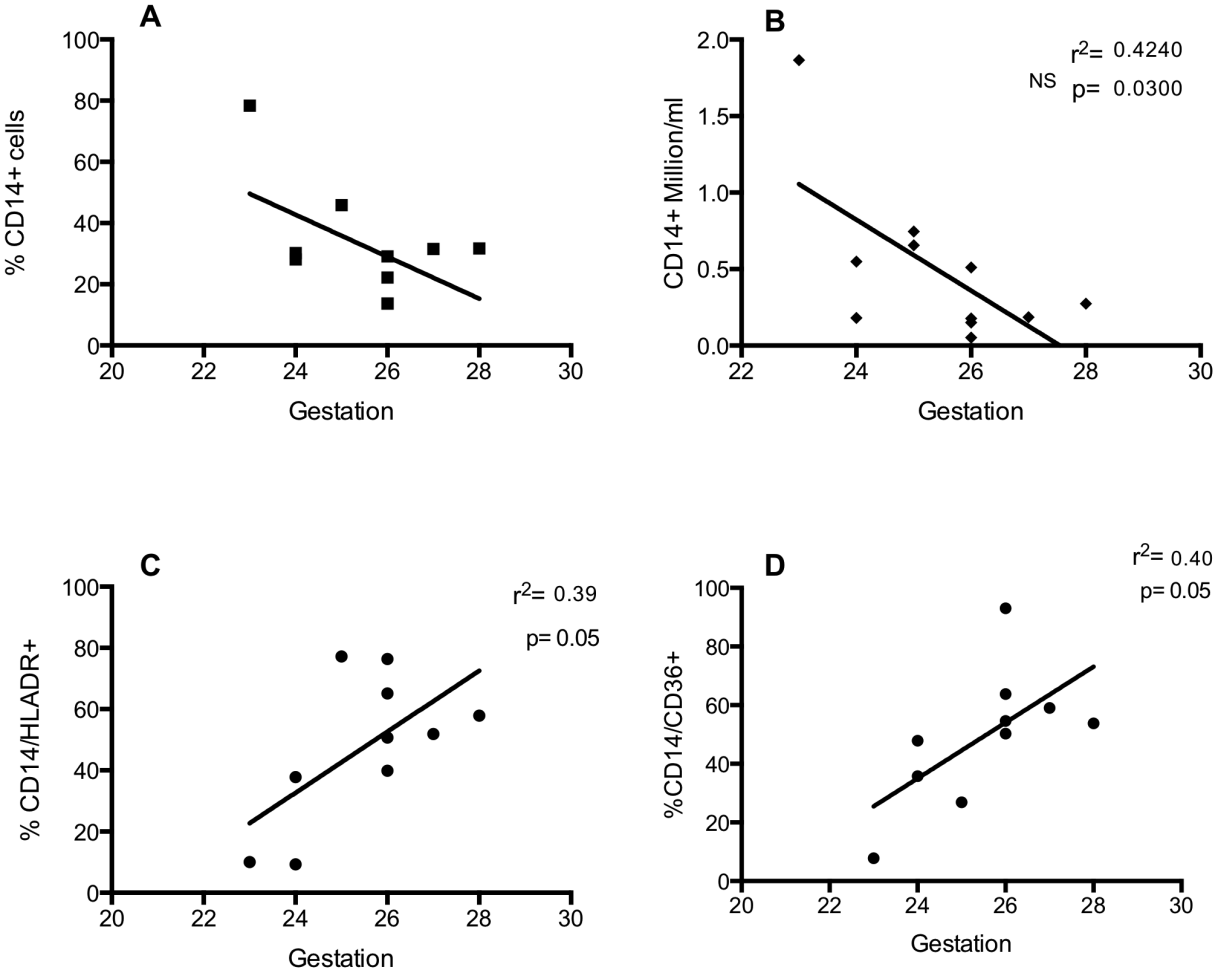
Early delivery is associated with increased numbers of macrophages and decreased populations of cells with an anti-inflammatory phenotype. Infants were ventilated and lavaged at 3 days of age and cells were stained with anti-CD14, anti-HLA-DR, anti-CD36 or matched isotype antibodies and analysed by flow cytometry. Data are expressed as percentage (A) or absolute numbers (B) of CD14+ events. Cells dual stained with CD14+/HLADR (C) or CD14+/CD36+ (D) are expressed as a percentage of all CD14+ events. Data are analysed by liner regression (n = 9–11 of which CLD∶RDS; 6–8∶3) and show R square and p values.

**Figure 5:**
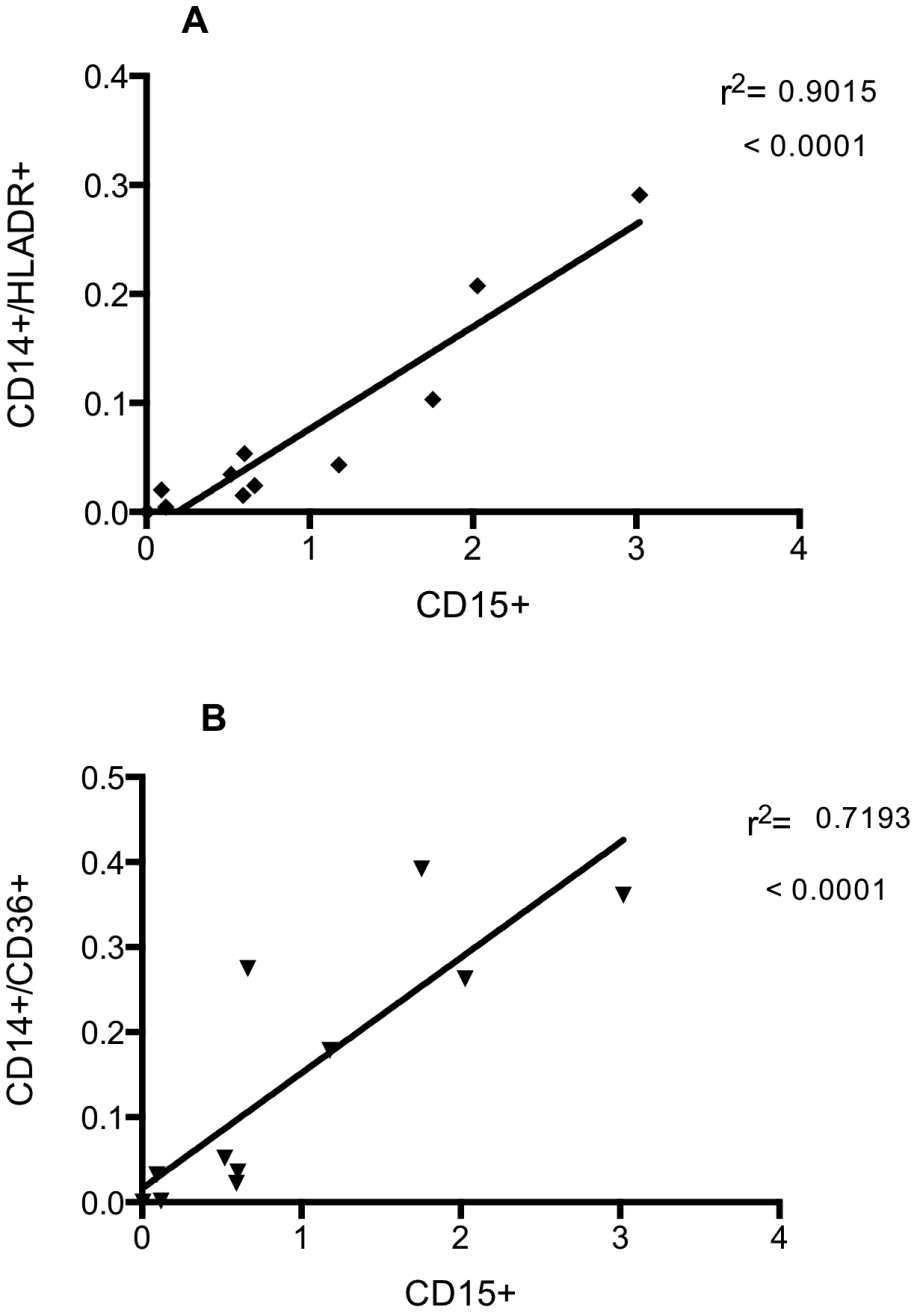
Numbers of neutrophils in the airway correlate with numbers of mature macrophages. Infants were ventilated and lavaged within 24-CD15, anti-CD14, anti-HLA-DR, anti-CD36 or matched isotype antibodies and analysed by flow cytometry. Data are expressed as absolute numbers of CD15+ events plotted against absolute numbers of CD14/CD36+ (A) or CD14/HLADR+ (B). Data are analysed by liner regression (n = 11 of which CLD∶RDS; 8∶3) and show R square and p values.

**Figure 6:**
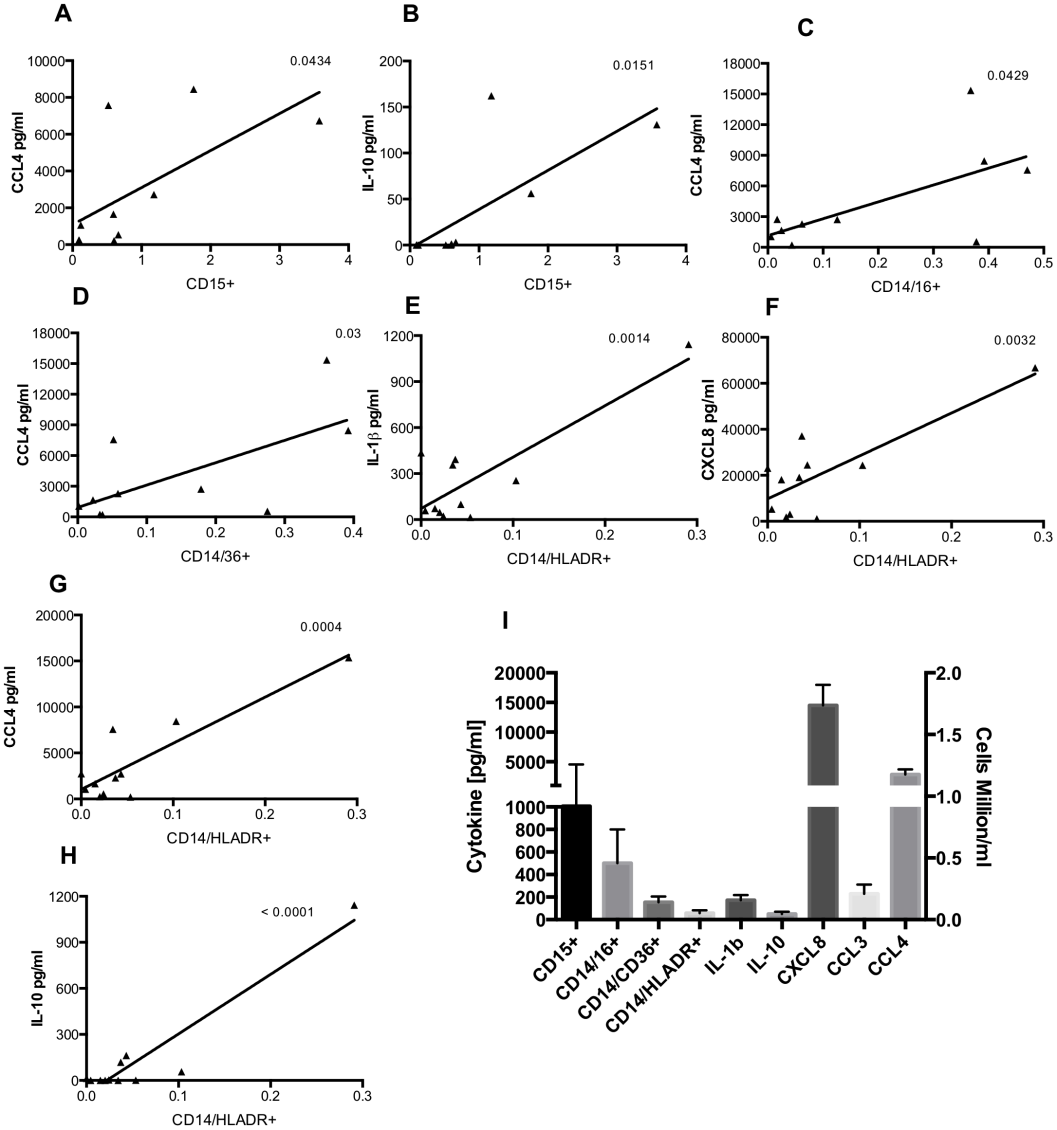
Cytokine levels correlate with cell type in preterm lavage. Preterm infants (CLD∶RDS; 8∶4) were ventilated and lavaged within 24 hours of birth and cells stained with combinations of anti-CD15 (A,B), anti-CD14(C–H), anti-CD16 (C), anti-CD36 (D), anti-HLA-DR (E–H), or matched isotype antibodies and analysed by flow cytometry. Cytokine bead arrays were carried out on cell-free lavage and the following cytokines were measured: IL-1β, CXCL8, CCL3, CCL4, and IL-10. Correlations of cytokine levels and absolute numbers of cells were analysed by linear regression and significant correlations are shown above. Panel I shows mean ± SEM absolute cell number (million/ml, right axis) and mean ± cytokine levels [pg/ml, left axis] for all samples.

To study the relationship between gestational age and the myeloid cell populations of the lung in the days following birth, some ventilated infants underwent ongoing sampling. Day 3 was chosen for further study as it allows observations to be made on recruitment and maturation of airway cells over a biologically relevant timescale, but which precedes extubation for many of the infants in this study. On day 3, the proportion of CD14^+^ cells was greater at low gestational ages, reaching statistical significance when analysing absolute numbers (figure 4A, B). There was a significant correlation between the proportion of CD14^+^ cells that were also positive for HLA-DR or CD36, and gestational age (figure 4C, D). To examine the relationship between the presence of neutrophils and macrophages in the immediate peri-natal period, absolute numbers of these cell populations were correlated. Absolute numbers of both CD14^+^/CD36^+^ and CD14^+^/HLA-DR^+^ macrophages were significantly correlated with numbers of neutrophils in BAL (figure 5A, B). Cytokines were also measured in BAL from preterm subjects by CBA and significant correlations were seen between absolute numbers of: CD15^+^ cells and CCL4 and IL-10; CD14^+^/CD16^+^ and CCL4; CD14/CD36^+^ and CCL3, CCL4; CD14/HLA-DR^+^ and IL-1β, CXCL8, CCL4 and IL-10 (table 2). Individual correlations of cell number and cytokine levels, where there is a statistically significant relationship, are shown in figure 6 (A–H). Mean cytokine levels ranged from 50.2±19.2 pg/ml for IL-10 to 14.5±3.5 ng/ml for CXCL8 (figure 6I).

**Table 2:** Cytokine levels correlate with cell type in preterm lavage.

	IL-1β		CXCL8		CCL3		CCL4		IL-10	
Cell type	p	r^2^	p	r^2^	p	r^2^	p	r^2^	p	r^2^
**CD15+**	-	-	-	-	-	-	0.04	0.42	0.02	0.54
**CD14/16+**	-	-	-	-	-	-	0.04	0.47	-	-
**CD14/36+**	-	-	-	-	-	-	0.03	0.44	-	-
**CD14/HLA-DR+**	0.001	0.69	0.003	0.63	-	-	0.0004	0.77	0.0001	0.89

Preterm infants were ventilated and lavaged within 24 hours of birth and cells stained with combinations of anti-CD15, anti-CD14, anti-CD16, anti-HLA-DR, anti-CD36 or matched isotype antibodies and analysed by flow cytometry. Cytokine bead arrays were carried out on cell-free lavage and the following cytokines were measured: IL-1β, CXCL8, CCL3, CCL4, and IL-10. Correlations of cytokine levels and absolute numbers of cells were analysed by linear regression and significant correlations are shown in the table.

**Figure 7:**
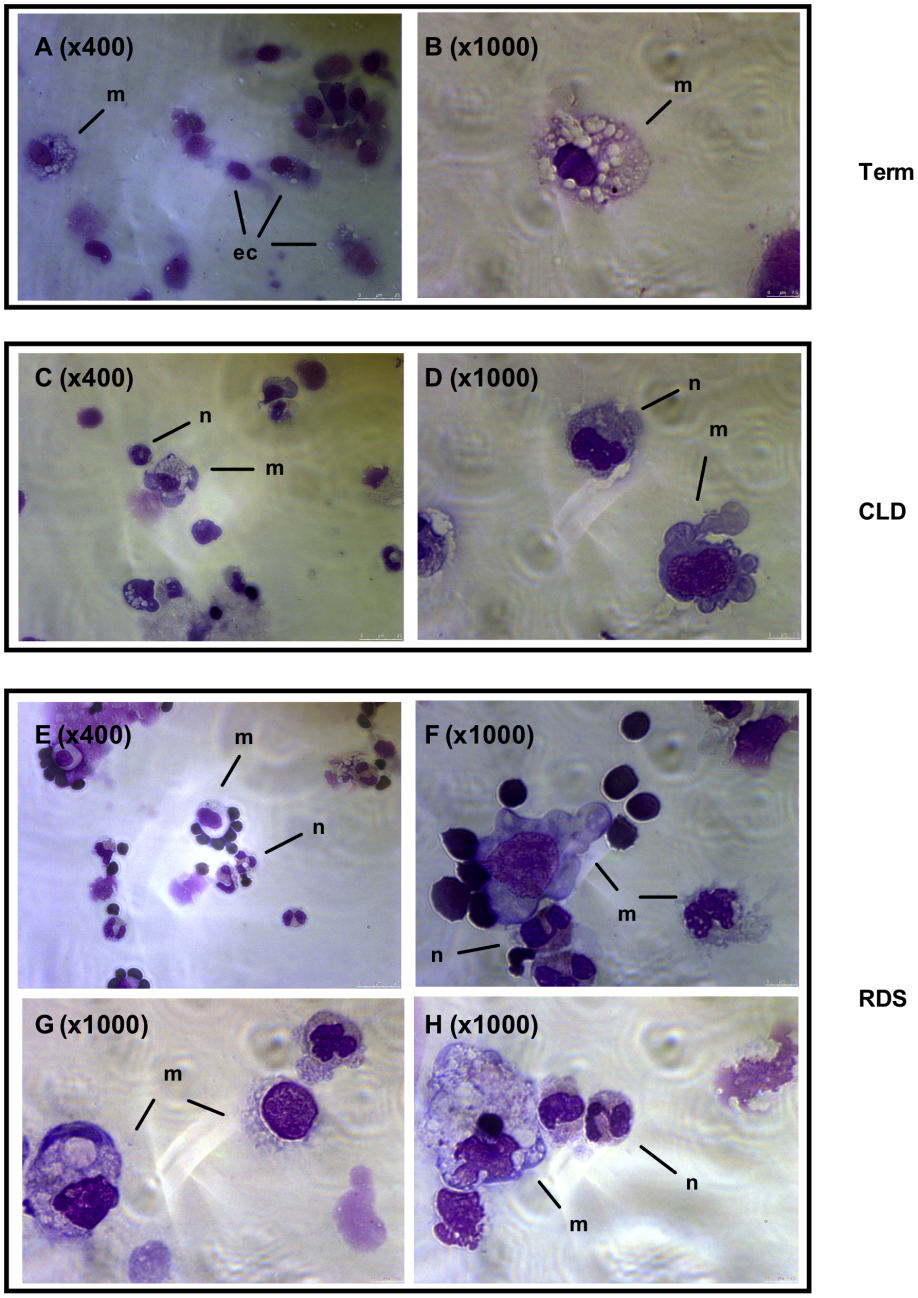
Morpholocial assessment of BAL cellularity. Infants were ventilated and lavaged within 24(A,B) and infants who developed CLD (C,D) or RDS (E–H) were studied. Images were viewed under ×400 (A,C,E) or ×1000 (B,D,E–H) lenses. Cell populations indicated as follows: m: macrophage, ec: epithelial cell, n: neutrophil Scale bar indicates 0–7.5 um in length.

**Figure 8:**
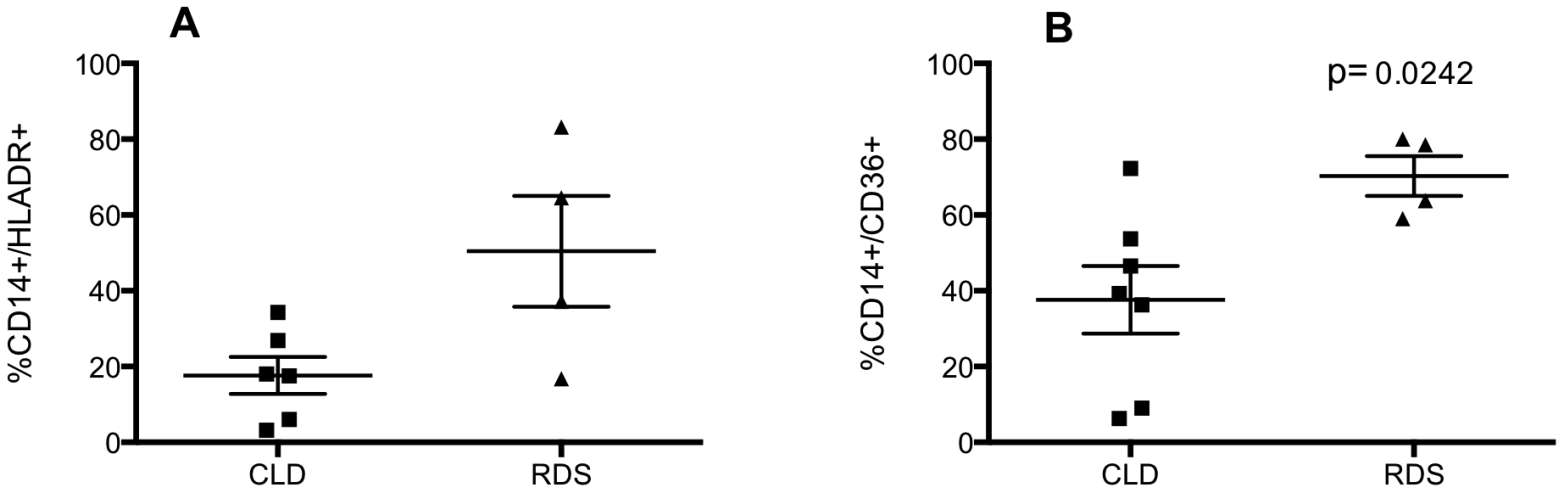
Inflammation resolution is associated with increased populations of anti-inflammatory macrophages. Infants were ventilated and lavaged within 24-CD14, anti-HLA-DR, anti-CD36 or matched isotype antibodies and analysed by flow cytometry. CD14^+^/HLA-DR^+^ and CD14^+^/CD36^+^ cells are expressed as percent of all macrophages (A, B). Statistical analyses were carried out by Mann Whitney test and compared samples collected from CLD (n = 6–7) to RDS (n = 4) infants.

Cytocentrifuge slides were made from BAL cells within 24 hours of birth and representative images are presented in figure 7. In keeping with figure 1, term infants who were ventilated for non-respiratory problems had few or no neutrophils and low numbers of macrophages, but the latter cells had the classical appearance of alveolar macrophages (figure 7A, B). Infants with CLD (figure 7C, D) and RDS (figure 7E–H) had more macrophages overall. A qualitative difference between macrophages from CLD and RDS was also observed, with macrophages isolated from RDS infants appearing larger and more mature. Phenotypic analysis of cells in these patient groups indicated that, as a percentage of macrophages, RDS infants had a higher percentage of CD36^+^ macrophages (figure 8B) yet the overall proportion of macrophages was similar between the two patient groups (data not shown). A trend for a greater proportion of HLA-DR^+^ macrophages was also seen in RDS compared to control groups but this was not statistically significant (figure 8A). There was no significant difference in percent of CD14+/CD16+ cells in the BAL of CLD versus RDS infants (data not shown).

## Discussion

CLD is a significant cause of morbidity and mortality in preterm infants, with symptoms continuing into adulthood at a great economic cost. Multiple factors occurring both pre- and post-natally contribute to the development of CLD including infection, ventilator induced lung injury, and a persistent inflammatory response. Current therapeutic strategies are of limited benefit, and mostly treat symptoms rather than the causative mechanisms, resulting in a significant and unmet need for novel and highly targeted medicines. To begin to address this issue, we must understand how normal cellular processes become dysregulated in the preterm lung, identifying both the relevant cell populations and the molecular events that lead to chronic inflammation and lung injury.

As a phenotypically heterogeneous cell population, the effector functions of alveolar macrophages vary with their differentiation status and functional polarisation. More differentiated alveolar macrophages are considered anti-inflammatory due to their abilities to secrete anti-inflammatory factors and participate in pathogen handling and efferocytosis [14]. We show preterm infants with resolving inflammatory lung injury have greater numbers of airway macrophages of a differentiated phenotype than infants with progressive inflammatory disease. We also show that prematurity is associated with increased populations of non-classical, pro-inflammatory monocytes-macrophages in the lung on the day of delivery. At day 3, we found infants born at a low gestational age are more likely to have greater numbers of CD14^+^ mononuclear phagocytes in the airway but fewer of these cells are functionally polarized, suggesting increased recruitment of monocytes or a failure to mature these cells in the lung. These findings suggest airway macrophage phenotype may be an important determinant of clinical outcome in neonatal inflammatory lung disease.

In humans, macrophages are thought mostly to differentiate post-natally with very few macrophages populating the sterile environment of the normal human lung at birth [15], [16], [17], [18]. *In vivo* studies show circulating monocytes appear from 20 weeks gestation [19] and macrophages are recoverable from the lung before birth [20], having matured under the influence of local factors including alveolar type 1 cells, bronchial epithelium, cytokines and surfactant [21]. Acquisition of secondary lysosomes is dependent on breathing air, suggesting other maturation processes do not occur until birth [22]. In rabbits, numbers of macrophages increase shortly before birth, continuing throughout the first month of life. Work using a murine lung explant model demonstrated not only that macrophages were present in the fetal lung but also that they were critical to lung immune responses [23], [24].

Macrophages containing apoptotic neutrophils have been visualized in BAL from preterm infants, supporting a functional role for these cells in the preterm infant lung [25]. Our current data show that both preterm and term infants have populations of macrophages in the lung at birth, as determined both by light microscopy and by co-expression of CD14 with CD36 or HLA-DR, presentational molecules with known immunoregulatory functions. HLA-DR is an antigen presentation receptor expressed on mature airway macrophages [26], . CD36 is a scavenger receptor expressed by both monocytes and macrophages, appearing at the peak of macrophage differentiation [28], [29], [30]. As well as facilitating bacterial phagocytosis, CD36 expression is associated with a macrophage phenotype that is more likely to efferocytose, and thus confers both a functional and anti-inflammatory phenotype upon the macrophage, and has been shown to play protective roles in inflammation [30], [31]. Flow cytometry data confirm these antigens as markers of differentiation, where CD36/HLA-DR^−^ macrophages appear as FSC/SSC low events, smaller and less granular cells, consistent with immature macrophages. CD36/HLA-DR^+^ macrophages however, have a higher FSC/SSC profile, consistent with that of mature tissue macrophages. Interestingly, preterm infants have a comparable proportion of HLA-DR^+^ macrophages to term infants, despite the developmental disadvantage of the former group, perhaps suggesting these cells have roles in immune surveillance and antigen presentation in utero. Other roles of macrophages in the sterile environment of the newborn lung may include ‘housekeeping’ roles such as removal of cellular debris, clearance of surfactant and remodeling and repair. Maintenance of macrophages in a detuned state facilitates homeostasis whereas inappropriate activation can arrest development (e.g BPD) and can lead to acute lung injury (e.g ARDS). It may also be beneficial to have immune defences already in place in anticipation of the potentially threatening event of birth.

The origins of mature airway macrophages at birth are unclear. They may be rapidly released from the pulmonary interstitial compartment at the time of birth [15]. It is also likely that mature airway macrophages can arise from the maturation of newly recruited monocytes upon breathing air, however this may occur over a number of days rather than the immediate perinatal period. Macrophages have been detected at autopsy in the lungs of human infants from 20 weeks gestation who died as a result of congenital pneumonia, suggesting that a significant inflammatory response pre- or peri-natally may trigger monocyte migration and macrophage maturation *in vivo* [16]. There is other evidence that the lung can be primed even before birth, for example in chorioamnionitis, which is associated with the development of CLD and may also influence the phenotype of cell populations in the lung [32], [33]. The presence of 16S ribosomal RNA was detected in some of the BAL samples (data not shown) but subject numbers did not allow us to probe for a significant relationship between infection and macrophage phenotype. It is also worth noting that corticosteroid treatment is another potential influence on cell maturation and function in utero, however, sample size and differences in the type and duration of treatment meant that we were unable to investigate a possible role for corticosteroids in this study.

In adults following acute pulmonary inflammatory stress, numbers of alveolar macrophages decrease and are replaced by macrophages of a more immature phenotype, possibly by rapid recruitment of monocytes from the vascular compartment [34]. In our study, day 3 BAL shows an inverse correlation between gestational age and CD14^+^ cells in the lung, yet infants of a low gestational age have, as a proportion of these CD14^+^ cells, fewer mature macrophages. These data may indicate a greater stimulus for monocyte recruitment into the lung and/or a possible failure of maturation in the more preterm individuals. This idea is in accordance with studies that show decreased HLA-DR expression on monocytes isolated from cord or venous blood from infants of a low gestational age compared with less preterm infants [35], [36], suggesting that the process of monocyte maturation is ongoing during gestation. Moreover, this phenomenon may continue into childhood, since macrophage HLA-DR expression is greater in children over compared to those under the age of two years [37]. Studies in mice have shown newly recruited peripheral blood monocytes and not alveolar macrophages are responsible for later-phase neutrophil migration to the lung following inflammatory stimulus, indicating immature macrophages are more likely to perpetuate on-going inflammation [38]. Thus very preterm infants may have a failure in the ongoing maturation process of tissue macrophages, which may in turn impair functional responses of these cells and increase the susceptibility of preterm infants to infection and inflammatory disease [17], [39], [40], [41].

The importance of the mononuclear cell phenotype in the context of inflammation has also been demonstrated in the form of CD14+/CD16+ populations, also referred to as ‘non-classical’ monocytes [42]. CD14+/CD16+ monocytes are highly synthetic cells that secrete pro-inflammatory cytokines and are a source of alveolar macrophages in the mouse [43]. Residing in the marginal pool, they rapidly extravasate into the tissue and become elevated both in the circulation and at inflammatory sites during inflammatory disease [12], [44], [45], [46]. Increased numbers of non-classical monocytes have been demonstrated in various inflammatory diseases including chronic liver disease, and these cells have an augmented inflammatory output both *in vitro* and *in vivo* [12]. Our data show significantly greater CD14+/CD16+ macrophages in preterm infants compared to term infants on the day of delivery. This difference may represent recruitment of increased numbers of circulating CD14+/CD16+ monocytes although further studies phenotyping peripheral blood monocytes to determine whether an increase in the tissue pool of non-classical macrophages are derived from greater numbers in the circulation, would be required to corroborate this. Since non-classical monocytes are capable of enhanced generation of pro-inflammatory cytokines, it is possible that this population may contribute to the inflammation observed in the lungs of preterm newborns.

We also examined the relationship between neutrophils, and macrophages expressing HLA-DR or CD36, which as a significant source of cytokines and chemokines may co-ordinate the recruitment of other cells. A strong correlation between mature macrophage and neutrophil numbers is seen. This could reflect a role for one of these cell types in the recruitment of the other, or simply that individual infants are more likely to globally recruit cells than others. Our preliminary study of relationships between BAL cytokine levels and different cell phenotypes suggests the former explanation, where The former explanation is more probable since CBA analysis of BAL also indicates a strong correlation between individual cell populations and cytokine levels at preterm birth, where in particular, HLA-DR^+^ macrophages are associated with IL-1β, CXCL-8, CCL4 and IL-10 levels, suggesting these cell types may be an important source of these cytokines [47]. HLA-DR expression correlates with increased cytokine production in monocytes [48] and in our study, the association with cytokine generation may reflect a more mature functionality of this macrophage population. Although CD36^+^ and HLA-DR^+^ macrophages represent mature phenotypes, they may differ in their cytokine output in the tissue. CD36^+^ macrophages are able to take part in efferocytosis, which is known to be associated with the production of anti-inflammatory cytokines by macrophages [28], [49], [50]. This may in part explain why the number of CD36^+^ macrophages does not correlate with pro-inflammatory cytokines such as IL-1β and CXCL8. Unexpectedly, CD36^+^ macrophage numbers are not associated with IL-10 in this study, a key macrophage anti-inflammatory cytokine.

We show that infants with progressive inflammatory disease (CLD) have fewer mature airway macrophages in the immediate peri-natal period compared to infants with resolving disease (RDS) suggesting phenotyping macrophages in ventilated infants may be a predictive marker of disease outcome allowing the stratification of treatment based on risk. Based on these findings, we speculate that the presence of mature airway macrophages may drive inflammation resolution, or indeed that the process of inflammation resolution may drive macrophage maturity. In support of this, we have previously shown an increase in total macrophages in BAL from infants with RDS compared to those with CLD, as determined morphologically [2]. Morphological data from this current study support the presence of mature macrophages in the lung of infants with RDS, illustrated by the appearance of large mononucleated cells with a high cytoplasm to nucleus ratio.

CD36 facilitates efferocytosis by macrophages, driving the resolution of inflammation [30], [51], and may in the context of neonatal inflammation, contribute to the clearance of tissue neutrophils and therefore promote inflammation resolution. Increased CD36^+^ macrophages in RDS infants compared to CLD infants may reflect the greater need for efferocytosis due to the increased neutrophil burden in the RDS lung [2]. In addition, it is conceivable that macrophages of a more immature phenotype may drive ongoing inflammation in the tissue, both via the synthesis of chemokines and enhanced activation status. A role for alveolar macrophages in disease has been established by others, although the maturity status of macrophages in these studies was not explored. Fetal lung macrophages contribute to the development of CLD/BPD in mouse models by generating a lung inflammatory response [24] and the presence of CD68^+^ macrophages is associated with Wilson-Mikity syndrome in preterm infants [52]. A study exploring HLA-DR expression on circulating monocytes in neonates showed decreased expression of HLA-DR in preterm infants with inflammatory lung disease compared to healthy preterm infants [53], suggesting that HLA-DR may be an important factor in the development of CLD, although differences were not statistically significant in our study. Proving the cause and effect relationship between statistically significant yet small differences in cell types was beyond the scope of this study, and future work correlating macrophage phenotype with cellular function ex vivo would be of great benefit.

In summary, we have demonstrated an association between macrophage phenotypes and disease progression in the context of chronic lung disease of prematurity. Not only do these findings associate macrophage phenotype with disease outcome in the context of neonatal lung disease, but also suggest new therapeutic possibilities focusing on delivering treatments to mature macrophage populations in the lung in preterm infants at risk of CLD. Furthermore, ex vivo phenotyping studies of ventilated preterm babies at birth may allow the stratification of risk in this patient group and enable treatment strategies to be tailored according to likely disease prognosis. These findings may help develop new therapeutic strategies focused on macrophage maturation in this clinically important disease.
